# Diabetes Surveillance in Germany – Background, concept and prospects

**DOI:** 10.17886/RKI-GBE-2017-022

**Published:** 2017-03-15

**Authors:** Lars Gabrys, Christian Schmidt, Christin Heidemann, Jens Baumert, Yong Du, Rebecca Paprott, Andrea Teti, Ingrid-Katharina Wolf, Thomas Ziese, Christa Scheidt-Nave

**Affiliations:** Department for Epidemiology and Health Monitoring, Berlin, Germany

**Keywords:** DIABETES MELLITUS, HEALTH MONITORING, DIABETES SURVEILLANCE, HEALTH REPORTING, PREVENTION

## Abstract

Diabetes mellitus is a chronic disease that is associated with serious health problems and high costs. According to estimates gained from nationally representative health surveys conducted by the Robert Koch Institute (RKI), 4.6 million adults aged 18 to 79 suffer from diabetes in Germany. In addition, around 1.3 million adults have undetected diabetes. A surveillance system is currently being established at the RKI in order to gather the data sources available on diabetes in Germany and to provide reliable and comparable findings on time trends covering the frequency, progress of treatment, prevention and care of the disease. Next to identifying trends, diabetes surveillance also needs to detect differences in epidemiology that are related to social status or geographic region. Diabetes surveillance at the RKI is being undertaken in close cooperation with stakeholders involved in science, health-care provision, health policy and health-system self-governance. Furthermore, its progress is accompanied by an interdisciplinary scientific advisory board.

Diabetes surveillance involves the following key elements: 1) the development of a research-based conceptual framework that uses indicators to appropriately measure developments in the disease; 2) the establishment of standards for the use of existing data sources and the identification of barriers to data usage and gaps in the data; and 3) the implementation of focused health reporting that is geared towards the target group. In addition to policy consultations, diabetes surveillance must guarantee the provision of timely and continuous information to the public together with the Federal Agency for Health Education. The implementation of a diabetes surveillance in Germany should act as a model and serve as a basis with which to establish the surveillance of other non-communicable diseases.

In principle, indicator-based diabetes monitoring at the population level can be viewed as providing the body for evidence-based policy consultation and focused health policy. In turn, this should enable the implementation of effective disease prevention measures and high-quality care for all groups within the population.

## 1. Background and aims

**Info box 1: The goals of the St Vincent Declaration (1989) [45]**

► Reduce new diabetes-related blindness by a third or more
► Reduce the frequency of diabetes-related terminal kidney failure
► Reduce the number of amputations due to diabetes-related gangrene by at least one half
► Reduce morbidity and mortality due to coronary heart disease in people with diabetes via intensive programmes aimed at reducing risk factors
► Normal pregnancy outcomes in diabetic patients and a similar rate of complications to those found in non-diabetic women

Diabetes mellitus covers a group of diseases that are characterised by a permanent increase in blood glucose concentrations. Diabetes is caused by a disorder of insulin secretion, reduced insulin sensitivity (insulin resistance) or a combination of both factors. There are two main forms of the disease: type 1 diabetes mellitus is an autoimmune disease caused by impaired insulin secretion resulting from the destruction of islet cells in the pancreas. By contrast, insulin resistance is the main factor in type 2 diabetes mellitus. Alongside a genetic predisposition, lifestyle factors play a crucial role in developing type 2 diabetes. In Germany, estimates from nationally representative health surveys conducted by the Robert Koch Institute (RKI) demonstrate that 4.6 million adults aged 18 to 79 have diabetes. In addition, around 1.3 million adults have undetected diabetes [1]. Current figures on the prevalence of diabetes in Germany as well as regional frequency distributions can be found in a separate article in this issue [2].

According to the latest research, approximately 90% of cases of the disease are type 2 diabetes mellitus. Men generally demonstrate a higher lifetime prevalence of diabetes in epidemiological studies than women [3]. In addition, a lower socioeconomic status is strongly linked to an increased disease prevalence [4]. Gestational diabetes is a special form of diabetes mellitus caused by insulin resistance which generally recedes after pregnancy; however, women with this form of the disease have a significantly higher risk of developing type 2 diabetes mellitus in later life [5, 6]. Importantly, if diabetes goes undetected or remains inadequately treated, it can cause life-threatening metabolic imbalances. Chronically elevated blood glucose levels result in the damage of blood vessels and the peripheral nervous system [7]. As a consequence the risk of cardiovascular disease, renal dysfunction, retinal damage and diabetic foot syndrome is increased [8, 9]. In addition, diabetes also causes long-term complications such as heart attacks, stroke, chronic kidney failure, blindness and amputations of the feet. Finally, pregnant women who have pre-existing diabetes or who develop diabetes during pregnancy have an increased risk of adverse pregnancy outcomes [10].

However, diabetes is not just linked to adverse effects for the individuals concerned, but also to costs to society. On the one hand, these costs arise from the expenses incurred through treatment (direct costs) and, on the other hand, due to the aggregate loss of economic productivity caused by sufferers’ incapacity to work and premature retirement (indirect costs). The German Federal Statistical Office estimates that the medical expenses accrued due to diabetes amounted to €6.3 billion in 2008 alone [11]. Current estimates calculate the annual medical expenses associated with diabetes as amounting to €16.1 billion [12]. Medical costs for people with diabetes are therefore between 1.7 and 1.8 times higher than for people without the disease [12-14].

In 1989, the St Vincent Declaration was adopted at the international level as a means of reducing the secondary health problems and premature mortality associated with diabetes (see Info box 1). Efforts to improve the treatment of people with diabetes have also been undertaken at national level, with improvements to care being implemented at health-system level. Since the introduction of the National Disease Management Guidelines (NDMG) on type 2 diabetes mellitus in 2002, evidence-based support is available to help make decisions on medical care; NDMG are continuously updated to reflect the latest research [15]. In 2003, the Disease Management Programmes (DMP) were first implemented for type 2 diabetes mellitus and later on expanded to type 1. The aim of the DMP is to ensure that patient treatment is structured, and that treatment outcomes are reviewed in accordance with established therapy and quality objectives [16, 17]. Over the last few years, enrolment in the DMP has steadily increased: in 2015, around 4 million patients with diabetes mellitus were enrolled in the programmes [18]. In addition, in 2003, the national health goal ‘Type 2 diabetes mellitus: reduction of disease risk, early recognition and treatment of patients’ was adopted; it defined specific measures and led to the establishment of pilot schemes in health care practice [19]. Alongside scientific evidence from studies of care provision, trend analyses based on the RKI’s health data also suggest that the structural changes mentioned above have contributed towards an improved care of people with diabetes [20, 21].

**Info box 2: The definition of secondary data**

In contrast to primary data, secondary data are data that were not gathered for a pre-defined investigative or research interest or that are analysed in a manner that is different from the original reason the data was collected. Boosted by the development of storage and computing capacities, in recent years, process-produced, and routinely-collected information has been harnessed increasingly for evaluations in health research. This also applies to data gathered from the contributions to and range of services provided by statutory health insurers. These developments have led the term ‘routine data’ to become synonymous with the more established term ‘secondary data’. A comprehensive overview of the issues associated with secondary data can be found in the manual ‘Routinedaten im Gesundheitswesen’ [46].

The situation described above demonstrates that diverse data sources and information and numbers already exist to measure occurrence of diabetes and care. However, until now, Germany’s federalist and pluralist health system has measured diabetes using data obtained from different sources each with a specific research focus. As such, these analyses have been limited in scope, and were not necessarily even based on a sustainable data source. In addition, their findings are rarely comparable as they focus on a variety of time periods and define their indicators in different ways. This situation makes it difficult to provide timely, evidence-based policy advice, which by contrast need reliable and comparable measures of the developments in diabetes and diabetes care.

Despite the fact that diabetes mellitus has high public health relevance, a comprehensive and continuous analysis of the disease, its consequences, developments in risk and care and the potential for prevention, have not yet been established at the population level. This is due to the complex causes of the disease, but also because of the fragmented data collection being undertaken and the fact that current data is usually tied to a specific purpose, as stated above. In addition, existing barriers to the use of secondary data (see info box 2) for research into scientific issues still need to be identified and dismantled.

**Fig. 1:**
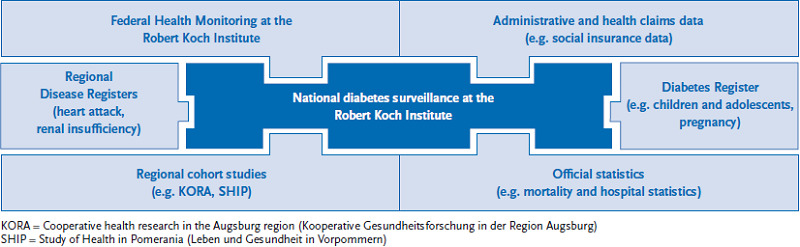
Data sources for national diabetes surveillance Source: own diagram

In the coming years, the RKI intends to establish a form of sustainable diabetes surveillance that is in line with the approach adopted by the World Health Organization (WHO) on the prevention and control of non-communicable diseases [22] and the associated recommendations on establishing effective surveillance mechanisms. The aim is to develop a form of diabetes surveillance that can be applied to the surveillance of other non-communicable diseases (as part of Non-communicable Disease Surveillance, NCD). This involves expanding the RKI’s existing health monitoring measures and integrating current data sources into an overall approach to diabetes surveillance (see Figure 1).

**Info box 3: Health monitoring and surveillance [47, 48]**

**Health monitoring**

► Periodically recurring collection and analysis of health data at the population level
► Comparable over time and internationally
► Scientific analyses and health monitoring for politics and the public

**Surveillance**

► Intensified monitoring of health problems that require increased vigilance
► Systematic analyses and current interpretations of continuously available health data
► ‘Data for Action’: policy advice, accompanying and evaluation research, development of measures

As is clear from info box 3, the development of health monitoring into a disease-specific form of surveillance means that, in addition to the recurring epidemiological description of the course of the disease and diabetes care, as derived from surveys based on interviews and examinations, timely analyses can be produced using regularly available routine data. Combining the primary data that the RKI already collects with secondary data sources enables a reliable and continuous data pool to be made available to health-policy decision-makers. Moreover, prompt demonstrations can then be provided of the specific areas where action needs to be taken, which can be followed up by targeted public health measures [23].

## 2. Project planning

The research project at the RKI aimed at developing a system of diabetes surveillance was initiated in December 2015. It is to extend over a four-year period and is funded by the Federal Ministry of Health.

The project is divided into three overlapping phases:

### Planning phase

► Review available data sources
► Define appropriate core indicators and gain a consensus about them
► Develop a conceptual framework

### Implementation phase

► Establish standards for merging information from different data sources
► Identify barriers to usage, and data gaps
► Conduct feasibility and comparative studies with research partners on the use of existing data sources

### Product phase

► Develop a model for regular, focused reporting
► Analyse the sustainability of the underlying data and the transferability of experiences and processes to other chronic diseases

From the planning phase to the beginning of the project, the focus has been placed on the development of a conceptual framework, the selection of indicators that appropriately capture the situation of diabetes in Germany, as well as an initial review of available data sources. The indicators need to reflect the evidence gained from indicators that have already been applied in the structured observation of diabetes mellitus in other countries [24-27] and international recommendations on the development of sustainable indicators [28-31].

During the implementation phase, which started parallel to the planning phase, analyses are conducted of existing data sources together with project partners to identify the ways in which the chosen indicators can be measured and to determine barriers to data usage and any remaining data gaps. During this phase, the RKI also examines how the health care data from the German statuatory health insurance funds (which is provided by the German Institute of Medical Documentation and Information – DIMDI) [32] can be continually integrated into diabetes surveillance.

During the product phase until the end of the project, a model form of focused diabetes reporting that is properly geared towards the target group is to be established as part of health reporting. The goal is to provide regular information to stakeholders in health policy, the public and science in the form of standardised analyses based on the indicators defined in the project about developments in diabetes in Germany.

An interdisciplinary project advisory board, which began its work in September 2016, accompanies project implementation [33]. The Scientific Advisory Board usually meets twice a year as part of a common board meeting.

Diabetes surveillance is also intended to provide a forum for international scientists and diabetes researchers from Germany, as well as patient representatives and health policy stakeholders to meet at conferences and workshops. On the one hand, the aim is to promote learning and knowledge transfer that can be incorporated into diabetes surveillance, but it is also aimed at improving networking between the people and institutions involved.

## 3. Current status

### 3.1 Developing a conceptual framework and defining core indicators

**Fig. 2:**
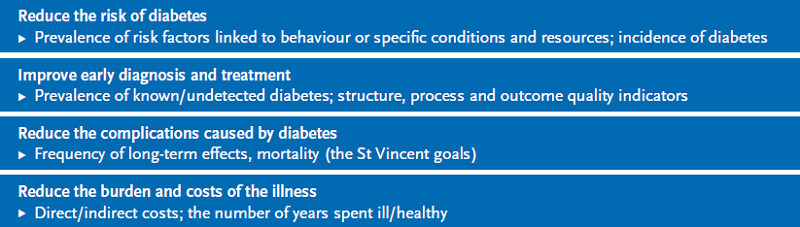
Relevant fields of action for the development of indicators in diabetes surveillance Source: own diagram

Four fields of action were defined in line with the health objective ‘Type 2 diabetes mellitus’ (which was adopted in 2003) and the Health Care Quality Indicators’ framework [34] developed by the Organisation for Economic Co-operation and Development (OECD). These fields were then assigned relevant concepts for the development of related indicators (see Figure 2).

The first step towards defining a set of core indicators involved a review of internationally established surveillance systems and diabetes registers. The review focused on the specifics of German health care and the German health care system. Potential single indicators were also compiled in parallel using a systematic literature study undertaken by the Institute for Applied Quality Improvement and Research in Health Care (AQUA). In a second step, an international expert workshop on indicator development took place in Berlin [35]; this was conducted after a structured review of indicators that had already been applied in research. The feedback from the expert panel was prepared both qualitatively and quantitatively and provided to the advisory board. In order to gain consensus on a core set of indicators, the indicators identified in this process will be evaluated in terms of relevance and feasibility using the Delphi method [36, 37].

**Fig. 3:**
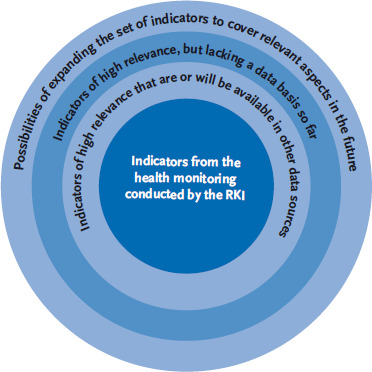
A schematic representation of the gradual extension of the indicator set in accordance with data availability Source: own diagram

It is unlikely that it will be possible to measure all indicators properly at the beginning of a study using existing data sources. Therefore, in addition to quality criteria, public health relevance, validity, clarity, sensitivity to change, comparability and health policy adaptability [30], data availability also represents an important criterion in the selection of indicators. Relevant indicators that cannot be properly captured using existing data sources are to be integrated into diabetes surveillance as part of a later step (see Figure 3).

The process of consensus used to select core indicators is to be completed in the first half of 2017, with the framework for diabetes surveillance due to be published soon afterwards.

Until now, very little data has existed for Germany on self-reported impairments, disease-specific knowledge and the informational needs of the people suffering from diabetes. For this reason, the RKI, in close cooperation with the Federal Centre for Health Education (BZgA), intends to conduct a separate nationally representative telephone survey of adults aged 18 and over on these issues. The survey’s findings are to be incorporated into an information and communication strategy being planned by the BZgA.

### 3.2 Conferences and workshops

The conceptual development of diabetes surveillance is supported by an intensive professional exchange conducted during expert workshops and specialist conferences that are regularly held during the duration of the project.

► Diabetes Register Conferences, in cooperation with the German Diabetes Association (DDG) and diabetesDE – German Diabetes Hilfe (launch event in Berlin, 22 April 2015)
  ▻ Definition of what is required of a national diabetes register/diabetes surveillance
  ▻ Possibilities and prospects of integrating diabetes registry data into a system of national diabetes surveillance
  ▻ Aims, data availability and data quality of diabetes surveillance in Germany
► Talks between the Federal (Federal Ministry of Health) and federal state level (launch event in Berlin, 22 July 2015)
  ▻ Coordination and cooperation with the federal states during regular workshops at the level of the Permanent Working Group of the Highest State Health Authorities (AOLG), AG Health Monitoring
  ▻ Regionalisation of diabetes reporting
► National expert workshops on the use of secondary data (launch event in Berlin, 7 December 2015)
  ▻ The use of health care data from the German Institute of Medical Documentation and Information (DIMDI) based on the Data Transparency Regulations (DaTraV)
  ▻ Integration, consolidation and use of additional secondary data sources in diabetes surveillance
► International expert workshops (launch event in Berlin, 11 July 2016) [35]
  ▻ Development and international comparability of indicator-based surveillance systems in Germany
  ▻ Best practice models for a national diabetes report

### 3.3 Review of data availability and usability

A key result of the diabetes register conferences listed above was that four methods projects on the use of secondary and registry data were initiated together with universities and other scientific institutions (see Table 1). The aim of these collaborations is to analyse the suitability of existing data sources for incorporation into diabetes surveillance and to close data gaps.

The projects presented here can be expected to provide important insights and indicators into developments in diabetes and diabetes care. For example, due to the low prevalence of type 1 diabetes and type 2 diabetes in children and adolescents, the RKI’s health surveys cannot provide any nationally representative findings on either form of the disease. In the future, these data gaps are to be closed by integrating data from the four existing registers covering type 1 diabetes in children and adolescents in Germany. In particular, the nationwide Diabetes Patient History Register (DPV) is focused on the continued observation of young patients into early adulthood and on the analysis of health care needs and the quality of care provision. Another project will provide data on gestational diabetes screening (which has been stipulated by law since 2015) and, thus, help improve the care of women with this form of the disease. Furthermore, potentially avoidable hospital admissions (AHA), in other words hospitalisations that could be avoided if care were better coordinated, constitute an important quality indicator of outpatient care, especially in the case of diabetes [38]. Moreover, in addition to survey and register data, increased use of secondary data sources is needed, in particular in order to be able to better represent aspects that are relevant to care using indicators of structural, process and outcome quality. To this end, the methods projects are developing suggestions as to which indicators can be represented using routine data from statutory health insurers and how this process can be better consolidated.

In the coming years, projects are also planned in cooperation with scientific project partners. The choice of projects depends on established criteria aimed at ensuring that their analyses and results can be integrated into diabetes surveillance.

## 4. Discussion

Unlike in some other countries, there is no continuous analyses or reporting aimed to summarize developments in diabetes mellitus or its associated health care provision being conducted in Germany. Data from other countries with an established surveillance system, such as the United States, have shown that active and systematic monitoring of developments in the disease have significantly reduced diabetes-and cardiovascular-related hospitalisations, in particular. Furthermore, appropriate preventive measures have also reduced the proportion of diabetes-related eye diseases and the rate of new occurrences of kidney disease [9, 39, 40]. Smaller countries with established disease registers, such as Denmark, Sweden and Scotland, have also noted improvements in diabetic care [41-43]. Although similar trends have been observed for Germany [20], the federal structure of the German healthcare system means that it has been difficult to develop a continuous and comparable analysis of health care provision over time.

The implementation of an indicator-based system of diabetes surveillance in Germany would finally make it possible to conduct comparative summarising analyses of the dynamics of diabetes as well as disease prevention and care provision over time. By developing a form of systematic diabetes surveillance, we can expect care structures and treatment approaches to be regularly evaluated in the future; this will make it possible to gauge the benefits they provide. Moreover, diabetes surveillance will also provide health policy-makers with evidence-based data that can be used to make decisions about the targeted allocation of funding aimed at improving care and diabetes prevention. However, in this regard, it is crucial that surveillance and health monitoring is geared towards the target group and that various disease prevention and care needs can be differentiated between so they can be represented according to socio-demographic and regional aspects over time. This is the only way of ensuring that health policy can provide a targeted and evidence-based response. This applies as much to primary preventive measures aimed at reducing the risk of diabetes as to secondary and tertiary prevention strategies aimed at improving diagnosis and treatment in order to prevent long-term complications. Close and continuous cooperation between epidemiology and care, as well as strong networking with health policy-makers, are of essential importance if the results of diabetes surveillance are to be of practical use. The experience gained outside of Germany demonstrates that the success of surveillance is dependent on the following factors:

► Agreement between all stakeholders from research, health practice and health policy about common goals and the consequences of action
► The application of defined quality criteria in the selection of indicators
► The possibility of developing stratified analyses that take into account socio-demographic and regional differences

As stated in the introduction, it is highly likely that adults with diabetes mellitus will develop other chronic diseases (co-morbidities). However, the majority of concomitant diseases and complications are linked to lifestyle-related risk factors such as obesity, a lack of physical activity and smoking. Therefore, diabetes surveillance needs to deploy a data pool and indicators that are relevant for the study of other non-communicable diseases. In Canada, it has been shown that diabetes surveillance can serve as a model for the surveillance of other chronic non-communicable diseases [44], as is recommended by the World Health Organization [22].

## 5. Conclusion

The implementation of diabetes surveillance should lead to the creation of a comprehensive and reliable data set for health-policy decision-making. Only when developments in the disease and diabetes care are systematically, periodically and regularly monitored and analysed, appropriate measures aimed at reducing the risk of diabetes and improving treatment can be evaluated and adapted. Improving the regional and sub-regional data pool and regionalised health monitoring is of particular importance here. In addition to providing policy advice, it is important to guarantee that information is made available to the public in a timely and continuous manner in cooperation with the German Federal Centre for Health Education. In the future, the surveillance of diabetes mellitus is to be used as a model with which to develop a similar system for other non-communicable diseases; this process will also involve striving for close international cooperation.

## Key statements

- The aim of the diabetes surveillance is to provide a sustainable data-driven decision-making basis with which to make public health policy-decisions that acts as a model for the surveillance of other non-communicable diseases.
- Until now, a coordinated set of indicators is missing that could enable an expanded form of health monitoring (surveillance) to be conducted using suitable data sources.
- A diabetes surveillance system covering Germany is being established at the Robert Koch Institute in close cooperation with stakeholders from science, care, health service self-administration and health policy.
- Due to the high disease frequency and the burden caused by diabetes, continuous observation and analysis of developments associated with the disease and its care are needed.

## Figures and Tables

**Table 1:** Projects for the use of available data sources in diabetes surveillance

**The harmonisation and correlation of register data**	
Project aims	► Prevalence estimates of type 1 diabetes in adults ► Prevalence estimates of type 2 diabetes in adolescents (11-17 years)
Data source(s)	► Child Diabetes Register DIARY (Diabetes Incidence Registry) ► Diabetes Register North Rhine-Westphalia ► Saxony’s Diabetes Register ► Diabetes Patients Progress Documentation (DPV-Register)
Project partners	► University of Ulm ► University of Tübingen ► Technical University Dresden ► German Diabetes Centre (DDZ), Düsseldorf
**Measuring the quality of care using routine data**	
Project aims	► Definition of core indicators measuring care quality ► Determination of a minimum data set
Data source(s)	► Routine data from AOK (a large statuatory health insurance company) Baden-Württemberg
Project partners	► University of Heidelberg ► Institute for Applied Quality Improvement and Research in Health Care (AQUA) Göttingen
**Expansion of data on quality of care in cases of gestational diabetes**	
Project aims	► Expansion of the gestational diabetes register and the development of a pilot region (North Rhine) ► Analysis of the quality of care provided to women with gestational diabetes ► Analysis of gestational diabetes screening
Data source(s)	► Gestational diabetes register (GestDiab)
Project partners	► Scientific Institute of established Diabetologists (winDiab)
**Surveillance of potentially avoidable hospital admissions (AHA) in cases of diabetes mellitus**	
Project aims	► Definition of relevant indicators for analysis of AHA in cases of diabetes mellitus ► Calculation of population-based rates of AHA ► Setting up of a time series 2005-2014
Data source(s)	► Statistics on Diagnosis Related Groups (DRG) (Federal Statistical Office)
Project partners	► Hochschule Niederrhein
